# Transmural Autonomic Regulation of Cardiac Contractility at the Intact Heart Level

**DOI:** 10.3389/fphys.2019.00773

**Published:** 2019-07-03

**Authors:** Yuriana Aguilar-Sanchez, Ainhoa Rodriguez de Yurre, Mariana Argenziano, Ariel L. Escobar, Josefina Ramos-Franco

**Affiliations:** ^1^Department of Physiology and Biophysics, School of Medicine, Rush University Medical Center, Chicago, IL, United States; ^2^Laboratorio de Cardio Inmunologia, Instituto de Biofisica Carlos Chagas Filho, Rio de Janeiro, Brazil; ^3^Division of Human Genetics, Children’s Hospital of Philadelphia, Philadelphia, PA, United States; ^4^Department of Bioengineering, School of Engineering, University of California, Merced, Merced, CA, United States

**Keywords:** mouse ventricle, endocardium, epicardium, adrenergic, cholinergic, whole heart

## Abstract

The relationship between cardiac excitability and contractility depends on when Ca^2+^ influx occurs during the ventricular action potential (AP). In mammals, it is accepted that Ca^2+^ influx through the L-type Ca^2+^ channels occurs during AP phase 2. However, in murine models, experimental evidence shows Ca^2+^ influx takes place during phase 1. Interestingly, Ca^2+^ influx that activates contraction is highly regulated by the autonomic nervous system. Indeed, autonomic regulation exerts multiple effects on Ca^2+^ handling and cardiac electrophysiology. In this paper, we explore autonomic regulation in endocardial and epicardial layers of intact beating mice hearts to evaluate their role on cardiac excitability and contractility. We hypothesize that in mouse cardiac ventricles the influx of Ca^2+^ that triggers excitation–contraction coupling (ECC) does not occur during phase 2. Using pulsed local field fluorescence microscopy and loose patch photolysis, we show sympathetic stimulation by isoproterenol increased the amplitude of Ca^2+^ transients in both layers. This increase in contractility was driven by an increase in amplitude and duration of the L-type Ca^2+^ current during phase 1. Interestingly, the β-adrenergic increase of Ca^2+^ influx slowed the repolarization of phase 1, suggesting a competition between Ca^2+^ and K^+^ currents during this phase. In addition, cAMP activated L-type Ca^2+^ currents before SR Ca^2+^ release activated the Na^+^-Ca^2+^ exchanger currents, indicating Ca_v_1.2 channels are the initial target of PKA phosphorylation. In contrast, parasympathetic stimulation by carbachol did not have a substantial effect on amplitude and kinetics of endocardial and epicardial Ca^2+^ transients. However, carbachol transiently decreased the duration of the AP late phase 2 repolarization. The carbachol-induced shortening of phase 2 did not have a considerable effect on ventricular pressure and systolic Ca^2+^ dynamics. Interestingly, blockade of muscarinic receptors by atropine prolonged the duration of phase 2 indicating that, in isolated hearts, there is an intrinsic release of acetylcholine. In addition, the acceleration of repolarization induced by carbachol was blocked by the acetylcholine-mediated K^+^ current inhibition. Our results reveal the transmural ramifications of autonomic regulation in intact mice hearts and support our hypothesis that Ca^2+^ influx that triggers ECC occurs in AP phase 1 and not in phase 2.

## Introduction

Cardiac excitability and contractility are tightly controlled by the autonomic nervous system (ANS) (Lee and Shideman, 1959; Katz, 1967; Lindemann and Watanabe, 1985; Cohn, 1989; Henning, 1992). The ANS comprises two antagonistic branches, the sympathetic and parasympathetic nervous systems. The sympathetic nervous system modulates the cardiac function through the local release of norepinephrine and systemic action of epinephrine that alter both, Ca^2+^dynamics (Lee and Shideman, 1959; Evans, 1986; Marks, 2013) and electrical activity of myocardial cells. At the cellular level, these catecholamines bind to β-adrenergic receptors (β-ARs) leading to the activation of adenylyl cyclase (AC) (Hildebrandt et al., 1983; Brum et al., 1984), through a *G*_s_ protein mechanism, to increase cAMP levels (Osterrieder et al., 1982). The cAMP activates PKA (Krebs, 1972; Hayes and Mayer, 1981), inducing the dissociation of the catalytic subunit to subsequently phosphorylate several key Ca^2+^ handling proteins. Some of these key proteins include the L-type Ca^2+^ channel (Collins et al., 1981; Osterrieder et al., 1982), ryanodine receptor (RyR2) (Suko et al., 1993; Valdivia et al., 1995) and phospholamban (PLN) (Mundiña de Weilenmann et al., 1987), a protein that inhibits the sarcoplasmic reticulum Ca^2+^ ATPase (SERCA).

The other branch, the parasympathetic nervous system, acts through the release of acetylcholine (ACh) from postganglionic cholinergic intracardiac neurons. The locally released ACh binds to muscarinic (M2) receptors which in turn inhibit AC through a *G*_i_ protein (Krebs, 1972). The AC inhibition favors low levels of cAMP, reducing the fraction of the activated PKA. The reduction of activated PKA will decrease the degree of phosphorylation of Ca^2+^ handling proteins. In atria, SA and AV nodes, the *G*_i_ protein-βγ subunits interact with an inward rectifying K^+^ channel increasing its open probability to produce an outward hyperpolarizing K^+^ current (*I*_KACh_) (Breitwieser and Szabo, 1988; Clapham and Kim, 1989; Kurachi et al., 1989; Wickman et al., 1999). Although the role of *I*_KACh_ in regulating the electrical properties of atria, SA and AV nodes have been extensively studied, the role of *I*_KACh_ during ventricular excitability is still obscure (Hiltunen et al., 2000; Rysevaite et al., 2011).

The influx of Ca^2+^ in ventricular myocytes is driven by the activation of L-type Ca^2+^ channels during an AP depolarization. In large mammals including humans, the prolongation of the AP plateau phase (phase 2) increases the time L-type Ca^2+^ channels are open leading to a sustained influx of Ca^2+^ into the myocyte. Small mammals like mice have a high metabolic demand. Thus, the heart needs to beat at higher rates to handle their metabolic needs. To cope with a higher heart rate, the mouse heart has developed a short duration ventricular AP. These characteristic kinetics promoted the idea that mouse ventricular AP lacks a plateau phase (phase 2) (Nerbonne and Kass, 2005; Dilly et al., 2006). Recently, our group presented experimental evidence showing that *in situ* mouse ventricular APs display a well-defined phase 2 (Ferreiro et al., 2012; Ramos-Franco et al., 2016). Interestingly, mouse AP phase 2 was more hyperpolarized than in large mammals (Kornyeyev et al., 2010; Valverde et al., 2010; Ferreiro et al., 2012) and it was driven by an influx of Na^+^ through the Na^+^-Ca^2+^ exchanger (NCX) (Ramos-Franco et al., 2016). However, up to now, it has not been possible to rule out the effect of AP phase 2 kinetics on intracellular Ca^2+^ dynamics in mouse hearts.

As sympathetic and parasympathetic drives impact the kinetics of both phase 1 and phase 2 (Litovsky and Antzelevitch, 1990) mimicking these autonomic regulations could be a physiological way to assess the role of these AP phases on cardiac contractility across the ventricular wall. Consequently, our goal is to test the hypothesis that in mouse cardiac ventricles the influx of Ca^2+^ that triggers excitation–contraction coupling (ECC) does not occur during phase 2. Our results reveal for the first time the transmural effects of autonomic regulation in intact mice hearts and confirm our previous observation that Ca^2+^ influx that triggers ECC occurs in the AP phase 1 and not in phase 2.

## Materials and Methods

### Heart Preparation

Experiments were conducted on 8-week-old, male Balb/c mice (Charles River Labs, Wilmington, MA, United States). Mice were maintained in accordance to the National Institutes of Health Guide for the Care and Use of Laboratory Animals (NIH Publication No. 85–23, Revised 1996) and the Institutional Animal Care and Use Committee guidelines of the University of California, Merced (Protocol # 2008–201). Mice were injected intraperitoneally with sodium heparin 15 min before euthanasia. Hearts were extracted by thoracotomy and perfused in a Langendorff apparatus with tyrode solution containing (in mM): 140 NaCl, 5.4 KCl, 2 CaCl_2_, 1 MgCl_2_, 0.33 NaPO_4_H_2_, 10 HEPES, and 10 glucose, pH 7.4 and equilibrated with 100% O_2_. Experiments were conducted at physiological temperatures of 35–37°C using a Peltier unit. The myosin ATPase inhibitor, blebbistatin (10 μM) was added to the tyrode solution to inhibit the heart’s mechanical activity. Blebbistatin was continuously perfused throughout the whole duration of the experiment to avoid introducing movement artifacts, breaking the glass electrode and photolability. All salts and drugs were obtained from Sigma (St. Louis, MO, United States). Tertiapin was purchased from Tocris (Minneapolis, MN, United States).

### Pressure Recordings

A new experimental approach, the micro-hemodynamic analyzer, was developed to measure left ventricular pressure in the whole intact heart (López Alarcón et al., 2019). A common device used to obtain this measurement consists of a small latex or plastic balloon coupled to a cannula that is introduced through the mitral valve. Usually, this balloon-cannula device is sutured to the atrial tissue and may cause damage. To avoid this problem, we introduced a tiny vitrectomy ophthalmic-valved trocar (Alcon, Fort Worth, TX, United States) in the apex of the left ventricle. The ventricular tissue, itself, provided a tight seal around the trocar avoiding any kind of leak from the left ventricular chamber. A 23-gage coupling valve was connected to the trocar, and the pressure at the outlet of the valve was measured with a solid-state integrated differential pressure transducer (Honeywell, Morris Plains, NJ, United States). The pressure transducer has an expanded dynamic range and can linearly measure pressures up to 200 mmHg. All the pressure measurements were performed under isotonic constant load conditions.

### Pulsed Local Field Fluorescence Microscopy

The pulsed local field fluorescence microscopy (PLFFM) technique has been previously used by our group (Mejía-Alvarez et al., 2003; Valverde et al., 2010; Ferreiro et al., 2012; Kornyeyev et al., 2012; Aguilar-Sanchez et al., 2017). Briefly, the PLFFM technique is capable of assessing physiological parameters by exciting exogenous probes present in the tissue and detecting the light emitted by these fluorescent indicators. The excitation (532 nm Yag laser) and emitted light propagate through a multimode fiber optic (200 μm diameter, 0.67 NA) placed on the surface of the intact heart. The emitted light then travels back through the multimode fiber, dichroic mirrors, and filters (610 nm) and is focused on an avalanche photodiode (Perkin Elmer, Waltham, MA, United States) with the aid of a microscope objective. The signal is digitized by an A/D converter (National Instruments) and acquired by a PC. Here, the PLFFM technique was modified to perform a comparative study, between endocardium and epicardium at the intact heart. We included a beam splitter which allowed us to use two fiber optics. This experimental arrangement facilitated the simultaneous recording of fluorescence from two distinct anatomical regions of the heart. For endocardial measurements, a small incision was made on the surface of the ventricular wall to gain access to the endocardial layer as previously described (Aguilar-Sanchez et al., 2017). Rhod-2 AM and Di-8-ANEPPS (Thermo Fisher Scientific, Waltham, MA, United States) were used to record intracellular Ca^2+^ transients and membrane potential, respectively. Rhod-2 AM and Di-8-ANEPPS were prepared with 20% Pluronic (Biotium, Hayward, CA, United States) in tyrode solution as previously described (Ferreiro et al., 2012; Aguilar-Sanchez et al., 2017). All dyes were sonicated for 20 min and then recirculated into the heart for 30 min at room temperature using peristaltic pumps.

### Electrical Recordings

Epicardial electrical recordings of the action potentials (APs) were obtained by means of sharp glass microelectrodes filled with 3M KCl that were connected to a high input impedance differential amplifier (WPI, Sarasota, FL, United States). Glass microelectrodes were fabricated with a micropipette puller (Flaming/Brown Micropipette Puller, Sutter Instrument Co., CA, United States) and had a resistance of 10–20 MΩ. All the AP recordings were performed in hearts that were externally paced at 6–7 Hz.

### Loose-Patch Photolysis

Our developed LPP technique was used to measure membrane currents during a triggered AP, at the intact heart level (Ramos-Franco et al., 2016). LPP combines three major elements: loose patch recordings, local field optical measurements, and a fiber optic laser-driven flash photolysis. The loose patch was performed using macro patch pipettes with a tip diameter of 250 μm. A fiber optic (200 μm) was placed in the interior to photolyze different drugs locally. This fiber delivered UV pulses to break photosensitive compounds on the membrane patch surface, making it possible to record the immediately resulting currents from 25 to 50 myocytes. Using this procedure, we were able to record membrane currents (capacitive and ionic) during an externally triggered AP before and after a UV flash. To dissect the UV-induced current, these sequential recordings were subtracted using pCLAMP. As we previously reported (Ramos-Franco et al., 2016), the capacitive current was not modified before and after the flash due to the electrotone imposed by the surrounding tissue on the small 200 μm UV flash site. Thus, the only current changing was the ionic current. We used the UV-sensitive compound, nifedipine (10 μM), such that the resulting currents were Ca^2+^-driven. The LPP was used to measure any changes in the Ca^2+^-driven currents in the epicardium under either continuous β-adrenergic or cholinergic stimulation. In addition, for transient β-adrenergic stimulation, we used the UV-sensitive caged cAMP (4,5-dimethoxy-2-nitrobenzyl adenosine 3′,5′-cyclic monophosphate).

### RNA Analysis

Hearts were perfused with RNAlater (Qiagen) to inhibit the RNAses. Then, left ventricular square pieces of 4 × 4 mm were included in agarose to facilitate slicing. Specimens were sliced in 200 μm sections using a vibratome (Leica VT1000S). For RNA extraction, individual slices were placed in a 1.5 mL Eppendorf containing 0.5 mL of Trizol reagent. Tissue was homogenized with an ultrasonic cell disruptor. The homogenate was incubated for 5 min at room temperature and 0.2 mL of chloroform per mL of Trizol was added. After 2–3 min of incubation, the sample was centrifuged at 12,000 × *g* 15 min 4°C. The clear phase was transferred to a new tube and 500 μL of isopropanol per mL of Trizol was added and centrifuged at 12,000 × g for 10 min at 4°C. The supernatant was removed and 1 mL of 75% ethanol per mL of Trizol was added and centrifuged at 7,500 × *g* for 5 min at 4°C. The dried pellet was dissolved in 30 μL of RNAse free water. For real time PCR, RNA was quantified (NanoDrop 1000 Spectrophotometer) and 2 μg was used for the reverse transcription reaction. The 2 μg of RNA was mixed with 1 μL of random primers (200 ng/μL) and water up to 12.5 μL. 7.5 μL of a MIX solution containing 4 μL of reaction buffer, 0.5 μL of RNAse free water, 2 μL dNTPs mix and 1 μL of RevertAid was added before the thermocycle cycles began. cDNA samples were diluted with water, 6.25 μL of Mix (FastStart Roche), 0.37 μL of forward primers, and 0.37 μL of reverse primers (see Supplementary Table 1). The reaction samples were placed in a 96-well plate and real time qPCR cycles were started. Finally, mRNA levels were normalized to GAPDH mRNA and analyzed by the Pfaffl method.

### Statistical Analysis

It is important to mention that in whole heart experiments there are two main sources of variance. First, the hearts are not identical between each other and second, although we are measuring Ca^2+^ transients and APs in the same region (the mid region of the left ventricle), it is impossible to perform the recordings in the same precise location between different hearts. Thus, the data is presented as the measured times with their standard error (SEM) in Tables and then as percentage change in Graphs, where data was normalized to the control values. To assess electrical changes, AP traces were evaluated at certain repolarization times. Specifically, the time it takes for the AP to reach 30%, half of phase 2, or 90% repolarization was assessed and referred to as APD30, APD half phase 2, or APD90, respectively. To evaluate the kinetics of the recorded Ca^2+^ transients, these were normalized between zero (minimum fluorescence) and one (maximum fluorescence). The kinetics parameters of the Ca^2+^ transients evaluated were the rise time (time for the transient to rise between 10% and 90%), time-to-peak (time for the transient to reach a maximum amplitude), half duration (time for half of the Ca^2+^ transient to be complete), and fall time (time taken for the transient to fall between 10% and 90% of the relaxation). Each recorded parameter for AP and Ca^2+^ transient kinetics, control and non-control experiments, were evaluated and normalized to the control values for each heart used. After this normalization, data was compiled and statistical analysis was performed. Data is presented as multiple measurements (*n*; dot cloud) recorded on different hearts (*N*) with the mean ± SEM (solid lines). The statistical significance was tested using a two-sample Kolmogorov–Smirnov test (OriginPro 2019). The difference was considered to be significant if the *p*-value was <0.01.

## Results

In the heart, both excitability and contractility are tightly controlled by the ANS. Although there is substantial information about sympathetic and parasympathetic regulation of the electrical and mechanical function, it is not clear how autonomically driven changes in the AP kinetics impact contractility in the mouse heart, which is often used as a model system.

**FIGURE 1:**
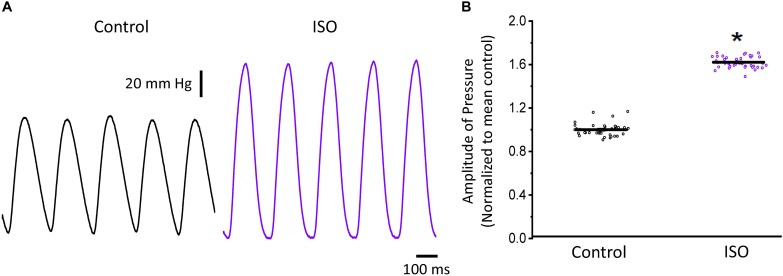
β-Adrenergic stimulation increases the left ventricular developed pressure. **(A)** Representative traces of the left ventricular pressure before (black) and after 500 nM isoproterenol (ISO; purple). **(B)** Data shows the change in the normalized amplitude of the developed pressure before and after isoproterenol. Data is presented as multiple measurements (*n*; dot cloud) recorded on different hearts (*N* = 4) with mean ± SEM (solid line) ^*^p < 0.01.

In a beating heart, the classical way to assess the degree of autonomic regulation is to evaluate the developed pressure during the cardiac cycle. Using intact perfused mouse hearts, we measured the effect of catecholamines in the developed pressure during the cardiac cycle. Figure 1 illustrates the time course of the developed pressure using the micro-hemodynamic analyzer (μHA) (López Alarcón et al., 2019). As was described in the “Materials and Methods” section, this newly developed method does not use an intraventricular balloon. Specifically, an ophthalmic-valved trocar was introduced through the apex of the left ventricle to gain access to the intra chamber pressure. The pressure at the outlet of the electro-valve was then measured with a solid-state integrated pressure transducer. This method permits an easy access to the ventricular chamber with no need of a more intrusive intraventricular balloon. Figure 1A illustrates how a sympathetic drive can increase the developed pressure in Langendorff perfused hearts. Indeed, when the hearts were coronary retro-perfused with 500 nM isoproterenol, we were able to record systolic pressures that were significantly larger than in control conditions. Figure 1B summarizes the pressure data obtained with our device, before and after the coronary perfusion with isoproterenol. The amplitudes of the developed pressures were normalized to the mean control. Isoproterenol induced a significant increase in the developed pressure (1 ± 0.06 for the control vs. 1.62 ± 0.05 for isoproterenol, *N* = 4 hearts *p* < 0.01), showing that the perfused hearts used here respond to a β-adrenergic drive.

**FIGURE 2:**
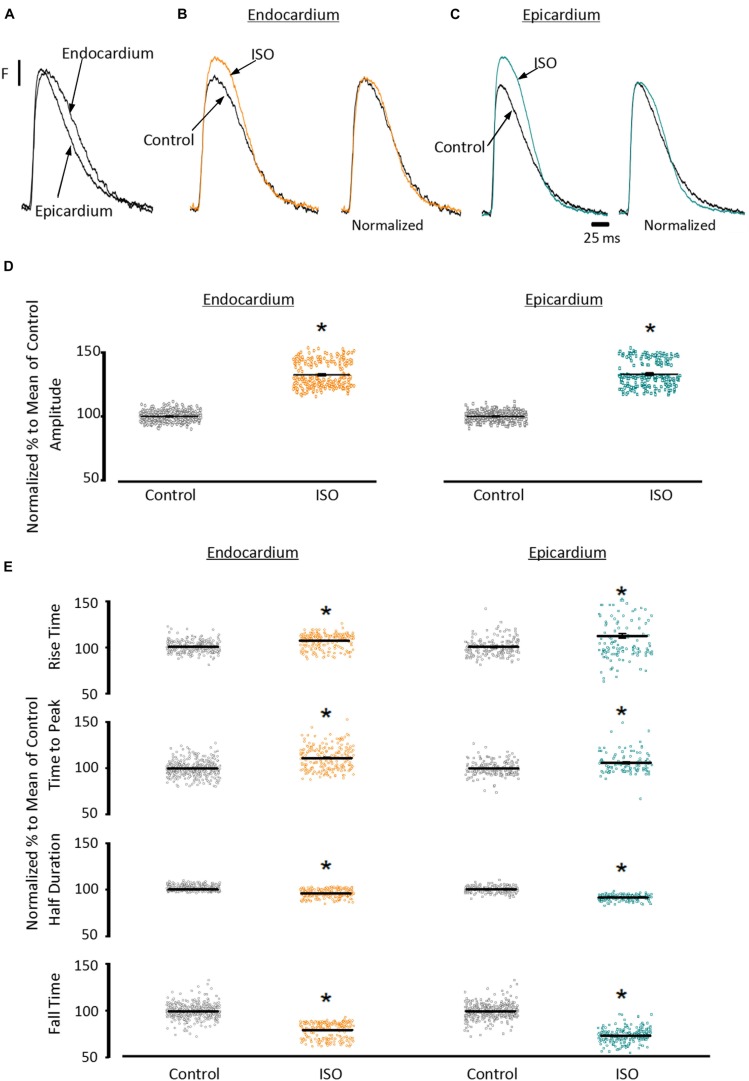
β-Adrenergic stimulation modulates Ca^2+^ transients in endocardium and epicardium. **(A)** Normalized fluorescent Ca^2+^ transients recorded, simultaneously, from endocardium and epicardium using Rhod-2 and the pulsed local field fluorescence microscopy technique. **(B)** Representative fluorescent traces of the endocardial Ca^2+^ transients before (black) and after 500 nM isoproterenol (ISO; orange). *Right panel*: Ca^2+^ transient traces normalized to their corresponding maximum fluorescence. **(C)** Representative Ca^2+^ transients recorded from the epicardium before (black) and after isoproterenol (cyan). *Right panel*: Ca^2+^ transients normalized to their maximum fluorescence. **(D)** Summary of the normalized amplitude of the Ca^2+^ transient changes, from the endocardium (circles) and epicardium (squares), before and after isoproterenol. **(E)** Data showing the kinetic changes in the normalized Ca^2+^ transients before and after isoproterenol. Means ± SEM are represented as the solid horizontal lines. ^*^p < 0.01, *N* = 4 hearts.

**FIGURE 3:**
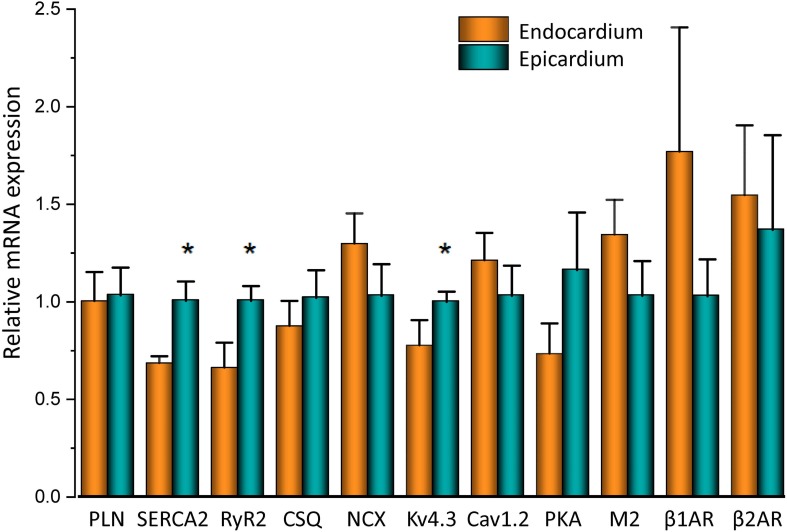
mRNA differences across the ventricular wall. Endocardial and epicardial mRNA quantification of the different proteins involved in Ca^2+^ signaling. mRNA levels (bars) were normalized to the housekeeping gene, GAPDH and expressed as mean ± SEM. *N* = 5. ^*^p < 0.05.

To assess the β-adrenergic regulation in the endocardial and epicardial layers, we examined the properties of Ca^2+^ transients in those particular regions using the PLFFM technique. Figure 2A illustrates normalized Ca^2+^ transients recorded simultaneously at the endocardial and epicardial layer. Perfusion of the hearts with 500 nM isoproterenol increased the amplitude of Ca^2+^ transients in both layers (Figures 2B,C). A summary of the effect of isoproterenol on the amplitude of the Ca^2+^ transients is presented in Figure 2D where it is possible to observe that there is a significant increase in both the endocardium (32.2 ± 0.83%) and the epicardium (32.9 ± 0.85%). This result is consistent with the effect of isoproterenol on the developed pressure as shown in Figure 1. Isoproterenol not only has an inotropic effect but also a lusitropic action. In Figures 2B,C it is possible to observe that the relaxation of the Ca^2+^ transients is faster in the presence of isoproterenol. Different kinetic parameters for endocardial and epicardial Ca^2+^ transients are compiled from 4 different hearts in Table 1 (measured times) and Figure 2E (normalized percentages). Isoproterenol induced significant changes in all the measured parameters of endocardial and epicardial Ca^2+^ transients. The rise time of the Ca^2+^ transients marginally increased (6.5 ± 0.83% for endocardium and 11.6 ± 3.1% for epicardium). As expected, the time to peak of the Ca^2+^ transients follow the same trend (11.1 ± 1.3% for endocardium and 5.9 ± 1.70% for epicardium). Isoproterenol induced a reduction in Ca^2+^ transients’ half duration. The half duration is statistically significant shorter in the presence of isoproterenol (5.9 ± 0.42% endocardium and 8.8 ± 0.45% epicardium). However, the changes in the fall time of the relaxation (20.3 ± 0.96% endocardium and 26.3 ± 0.92% epicardium) were larger than those observed in the half durations. The large effect of isoproterenol on the fall time compared to the half duration could be explained by the fact that isoproterenol not only has a positive lusitropic effect but also changes the morphology of the Ca^2+^ transients as shown in Figures 2B,C. The changes in fall time for the epicardial were significantly faster (*p* < 0.01) than those in the endocardial layer (Figure 2E, bottom panel). It is known that cardiac myocytes from the endocardial, midmyocardial and epicardial layers are characterized by molecular heterogeneities at the level of transcription and expression of proteins. Thus, the faster fall time observed in epicardium can be molecularly defined by a higher expression of SERCA in this layer. Indeed, when we assessed the levels of mRNA transcription by qPCR, there were significant changes in the expression of RyR2, SERCA and Kv4.3, a K^+^ channel responsible for *I*_to_ that consistently shows a higher expression in the ventricular epicardium (Figure 3). However, we did not find significant differences in the expression of L-type Ca^2+^ channels, NCX, PKA, M2 muscarinic receptors and β-ARs between both layers. Since the expression of both RyR2 and SERCA is unevenly distributed across the ventricular wall, we expected to have a differential response between endocardium and epicardium.

**TABLE 1:** Isoproterenol effects on Ca^2+^ transient kinetics and AP durations.

**Isoproterenol effects on Ca^2+^ transient kinetics**				
**Condition**	**RT (ms)**	**TP (ms)**	**HD (ms)**	**FT (ms)**
Endo Control	7.95 ± 0.05 (*n* = 269)	11.59 ± 0.07 (*n* = 376)	49.93 ± 0.49 (*n* = 390)	65.61 ± 0.40 (*n* = 426)
Endo Iso	7.96 ± 0.08 (*n* = 210)^*^	12.51 ± 0.08 (*n* = 199)^*^	49.20 ± 0.61 (*n* = 216)^*^	52.76 ± 0.70 (*n* = 215)^*^
Epi Control	6.78 ± 0.05 (*n* = 185)	11.70 ± 0.08 (*n* = 183)	46.83 ± 0.18 (*n* = 335)	69.30 ± 0.39 (*n* = 311)
Epi Iso	7.16 ± 0.13 (*n* = 130)^*^	12.11 ± 0.90 (*n* = 112)^*^	43.09 ± 0.31 (*n* = 233)^*^	49.79 ± 0.44 (*n* = 232)^*^

**Isoproterenol effects on AP durations**				
**Condition**	**APD30 (ms)**	**APD half phase 2 (ms)**	**APD90 (ms)**	

Endo Control	8.26 ± 0.13 (*n* = 143)	58.91 ± 0.27 (*n* = 89)	76.01 ± 0.66 (*n* = 90)	
Endo Iso	10.21 ± 0.16 (*n* = 141)^*^	54.54 ± 0.18 (*n* = 87)^*^	67.10 ± 0.33 (*n* = 88)^*^	
Epi Control	2.93 ± 0.03 (*n* = 236)	62.08 ± 0.30 (*n* = 90)	77.45 ± 0.54 (*n* = 90)	
Epi Iso	3.77 ± 0.04 (*n* = 280)^*^	54.43 ± 0.18 (*n* = 89)^*^	69.81 ± 0.45 (*n* = 88)^*^	

Endo, endocardium; Epi, epicardium; Iso, isoproterenol; RT, rise time; TP, time to peak; HD, half duration; FT, fall time. APD, AP durations measured at 30, half phase 2, and 90% repolarization. ^*^Kolmogorov–Smirnov test, *p* < 0.01, *N* = 4.

**FIGURE 4:**
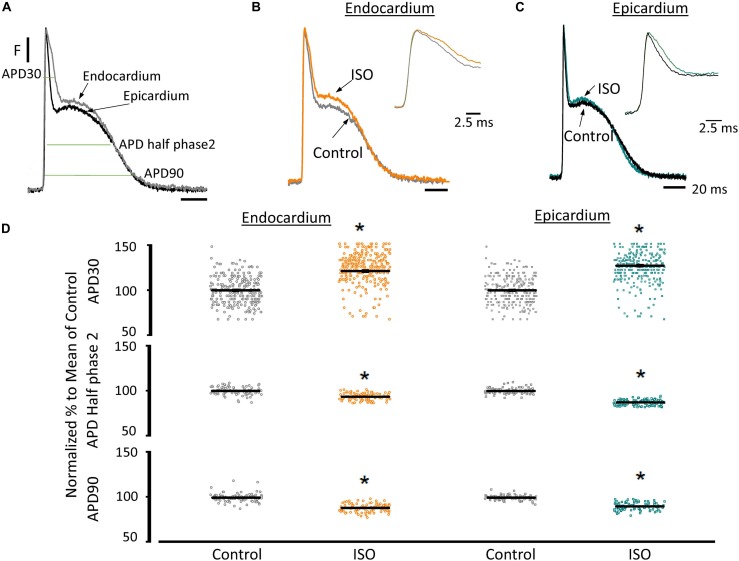
β-Adrenergic stimulation modifies AP morphology in endocardium and epicardium. **(A)** Normalized APs from the endocardium (gray) and epicardium (black) recorded optically using Di-8-ANEPPS and the pulsed local field fluorescence microscopy technique. The evaluated AP parameters are marked. **(B)** Normalized APs from the endocardium before (gray) and after perfusion with 500 nM isoproterenol (ISO; orange). *Inset*: Expanded view of AP phase 0 and 1 to visualize the isoproterenol effects. **(C)** Normalized APs recorded from the epicardium before (black) and after perfusion with 500 nM isoproterenol (cyan). *Inset*: Expanded view of AP phase 0 and 1 to visualize the isoproterenol effect. **(D)** Summary of the normalized APD30, APD half phase 2 and APD90 changes from the endocardium (circles) and epicardium (squares), before and after isoproterenol. Means ± SEM are represented as the solid horizontal lines. ^*^p < 0.01, *N* = 4 hearts.

Another remarkable difference that distinguishes the endocardial and epicardial layers is their electrical properties. One of the main differences is in the phase 1 repolarization of the AP due to the higher expression of Kv4.3 in the epicardial layer (Figure 3). Thus, we designed experiments to evaluate the hypothesis that β-adrenergic stimulation will differentially affect the time course of endocardial and epicardial APs. Figure 4A compares optically recorded ventricular APs in the endocardium and epicardium, where all phases are distinguished. The APs in both layers have a well-defined phase 2. However, the repolarization rate of phase 1 is faster in epicardium than in endocardium (Table 1). The perfusion with 500 nM isoproterenol produced changes in the AP morphology (Figures 4B,C). Unexpectedly, isoproterenol decreased the repolarization rate of phase 1, in both the endocardium (Figure 4B, inset) and the epicardium (Figure 4C, inset). Table 1 and Figure 4D summarizes different endocardial and epicardial AP parameters from four hearts. The most salient features of β-AR activation were an increase in the duration of phase 1 (APD30) and a shortening of the late phases of the AP (APD half phase 2 and APD90). Indeed, isoproterenol induced an increase in APD30 of 21.2 ± 2.40% in endocardium and 27.6 ± 2.18% in epicardium. As in other mammalian species (Litovsky and Antzelevitch, 1990), isoproterenol induced a shortening in APD half phase 2 duration in endocardium and epicardium, 6.9 ± 0.90% and 12.6 ± 0.59%, respectively. Isoproterenol also shortened APD90, 11.5 ± 0.96% in the endocardium and of 9.8 ± 0.73% in epicardium (Figure 4D).

**FIGURE 5:**
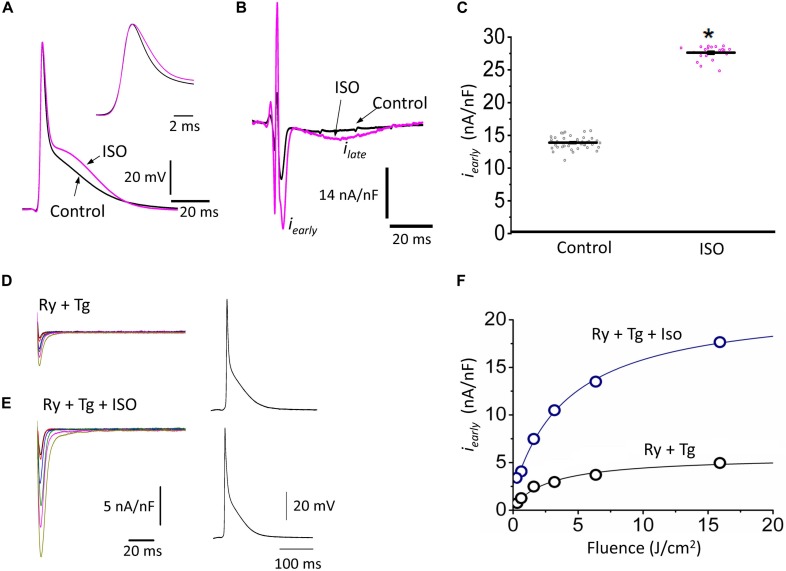
β-Adrenergic stimulation increases Ca^2+^-driven currents. **(A)** APs recorded from the epicardium using glass microelectrodes before (black) and after 500 nM isoproterenol (ISO; pink). *Inset*: Expanded view of the phase 0 and 1 showing the effects of isoproterenol on APD30. **(B)** Representative traces of Ca^2+^-driven currents recorded after the local photolysis of nifedipine using the Loose Patch Photolysis technique showing the *i_early_* and *i_late_* components upon β-adrenergic stimulation. **(C)** Data from the *i_early_* component, representing the L-type Ca^2+^ currents, before (gray) and after isoproterenol (pink). The means ± SEM are represented as the solid horizontal lines. ^*^p < 0.01, *N* = 4 hearts. **(D)** Representative traces of Ca^2+^-driven currents recorded consecutively at increasing UV energies (fluences) in control conditions in the presence of ryanodine (Ry, 10 μM) and thapsigargin (Tg, 2 μM). *Right panel*: Simultaneously recorded AP in the same conditions. **(E)** Ca^2+^-driven currents recorded consecutively at increasing UV energies (fluences) with isoproterenol in the presence of ryanodine (Ry, 10 μM) and thapsigargin (Tg, 2 μM). *Right panel*: Simultaneously recorded AP in the same conditions. **(F)** Peak values of *i_early_* as a function of the fluence, before (black) and after isoproterenol (blue) in the presence of Ry and Tg.

The decrease of the repolarization rate of phase 1 with isoproterenol can be attributed to an increase in the amplitude of L-type Ca^2+^ currents. We recently demonstrated that most of the Ca^2+^ influx that triggers Ca^2+^-induced Ca^2+^-release (CICR) occurs during phase 1 (Ramos-Franco et al., 2016) and that the influx of Ca^2+^ through L-type Ca^2+^ channels slows down the rate of repolarization during phase 1 (López Alarcón et al., 2019). To directly evaluate any changes in the L-type Ca^2+^ currents during isoproterenol perfusion, we used the novel Loose Patch Photolysis (LPP) technique. We simultaneously acquired Ca^2+^-driven currents with the corresponding APs before and after isoproterenol. In Figure 5A, the AP showed an increase in APD30 (inset) as well as in phase 2. However, due to the presence of nifedipine, this increase in APD30 is smaller than in its absence (Table 1 and Figure 4D). Figure 5B shows a typical experiment where Ca^2+^ currents, activated by the photolysis of nifedipine on the membrane patch surface (see “Materials and Methods” section for details), were recorded before and after the heart was perfused with isoproterenol. We observed a clear increase in the amplitude of the Ca^2+^-driven currents, displayed as an early fast component (*i_early_*) and a late slower component (*i_late_*). Unfortunately, the simultaneous measurement of Ca^2+^ driven currents in the endocardium and epicardium was not possible due to the size of the patch pipettes. To reach the endocardial layer with the patch pipette would require a large size hole that would result in electrical conduction alterations. Figure 5C shows the effect of isoproterenol on the amplitude of *i_early_* that increased from 13.9 ± 0.2 nA/nF to 27.6 ± 0.2 nA/nF. In a recent work (Ramos-Franco et al., 2016), we showed that Ca^2+^ released from the sarcoplasmic reticulum (SR), activated the NCX in the forward mode, resulting in *i_late_* (Ferreiro et al., 2012; Ramos-Franco et al., 2016). This *i_late_* was abolished (Figure 5D) when we blocked SR Ca^2+^ release with a combination of ryanodine and thapsigargin. The family of traces correspond to consecutive current recordings, where the Ca^2+^ currents were evoked locally by progressively photolyzing greater fractions of nifedipine by using UV laser pulses of increasing beam energy (fluence). To test the hypothesis that β-adrenergic stimulation directly increases the amplitude of the Ca^2+^ current occurring during phase 1, we performed an identical experiment to the one presented in Figure 5D but in the presence of isoproterenol (Figure 5E). We observed that isoproterenol dramatically increased the amplitude of *i_early_*. The simultaneous recording of the corresponding APs shows that isoproterenol only altered the kinetics of phase 1 (Figure 5E, right panel). Figure 5F shows the peak values of *i_early_* as a function of the fluence, before and after isoproterenol. It is possible to observe that *i_early_* saturates at high fluence values (>16 J/cm^2^) indicating that most of the nifedipine was photolyzed.

**FIGURE 6:**
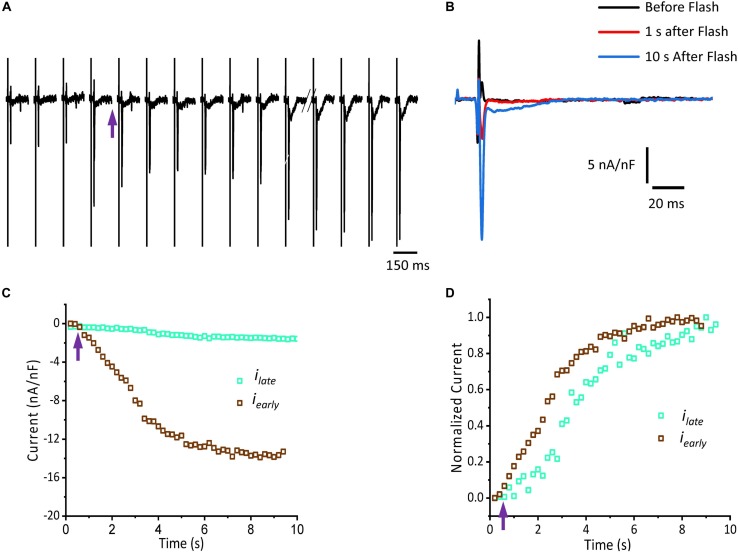
cAMP modulates the time course of Ca^2+^-driven currents. **(A)** Ionic currents recorded during consecutive cardiac cycles before and after the photolysis of caged cAMP. **(B)** Ionic currents induced by cAMP taken from **(A)** at the indicated time points (traces black, red, and blue) are overlapped to demonstrate the effect in amplitude for both *i_early_* and *i_late_* components. **(C)** Current amplitude peaks of *i_early_* and *i_late_* as a function of time before and after the photolysis of caged cAMP. **(D)** Normalized data from **(C)** re-plotted as a function of time. Purple arrow indicates the time of the UV pulse (cAMP uncaging).

Figure 5 shows that isoproterenol induced an increase in the amplitude of *i_early_*. It is well known that catecholamines activate PKA, which in turn leads to the phosphorylation of multiple targets involved in Ca^2+^ signaling (i.e., the L-type Ca^2+^ channels, RyR2 and PLN). In order to determine which target is phosphorylated first, the effect of catecholamines on the time course of Ca^2+^-driven currents was evaluated. We performed experiments in which we mimicked a transient β-adrenergic stimulation by photolyzing caged 3′,5′-cyclic adenosine monophosphate (cAMP). A typical response is illustrated in Figure 6A where upon immediate photolysis (purple arrow), both components of the Ca^2+^-driven current, *i_early_* and *i_late_*, began to increase over time. To better illustrate the changes of *i_early_* and *i_late_*, in Figure 6B, we overlapped the current traces at different time points: before, 1 s and 10 s after the uncaging. Plotting the amplitude peaks of *i_early_* and *i_late_* as a function of time during consecutive cardiac cycles, highlighted that both currents changed over time, but with a greater effect on *i_early_* (Figure 6C). When we normalized these currents (Figure 6D), it became apparent that *i_early_* also responded sooner to the transient β-adrenergic stimulation.

**FIGURE 7:**
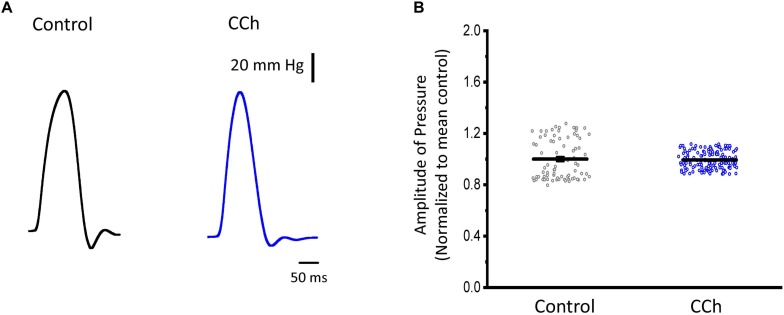
Cholinergic stimulation does not modify the left ventricular developed pressure. **(A)** Representative traces of the left ventricular pressure before (black) and after 5 μM carbachol (CCh; blue). **(B)** Data of the normalized amplitude of the developed pressure before and after carbachol. Means ± SEM are represented as the solid horizontal lines, *N* = 3 hearts.

Sympathetic stimulation is highly antagonized by the other branch of the ANS, the parasympathetic nervous system. Parasympathetic stimulation is thought to generally predominate over an existing sympathetic activation. Next, we evaluated the response of endocardium and epicardium to muscarinic cholinergic agonists. Figure 7A shows that upon perfusion with 5 μM carbachol, there were no measurable changes in the developed ventricular pressure. Our pressure data (Figure 7B) shows there was no significant differences before and after carbachol (0.99 ± 0.15 vs. 0.99 ± 0.07, *N* = 3 hearts). These results suggest that in the absence of a β-adrenergic stimulus, carbachol by itself does not induce a decrease in the developed ventricular pressure during the cardiac cycle.

**FIGURE 8:**
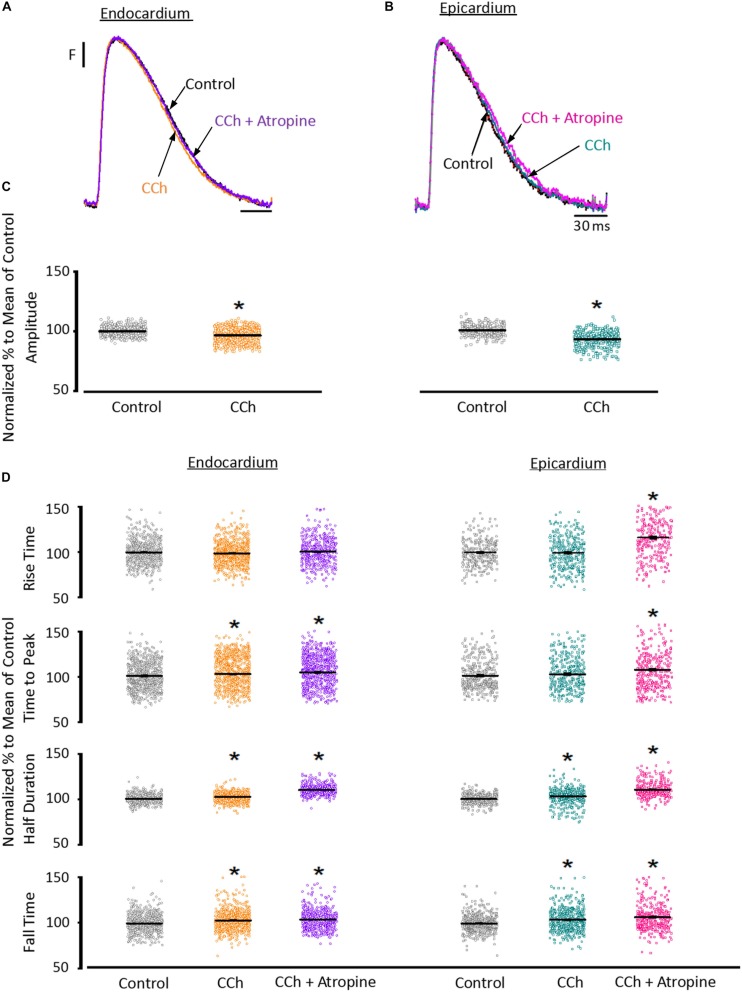
Cholinergic stimulation has a low impact on the Ca^2+^ transients in endocardium and epicardium. **(A)** Normalized fluorescent Ca^2+^ transients recorded, simultaneously, from endocardium and **(B)** epicardium using Rhod-2 and the pulsed local field fluorescence microscopy technique before and after 5 μM carbachol (CCh) and in the presence of both carbachol and 40 μM atropine. Overlapping Ca^2+^ transients were normalized to their maximum fluorescence. **(C)** Summary of the normalized amplitude of the Ca^2+^ transient changes, from the endocardium (circles) and epicardium (squares), before and after carbachol. **(D)** Data showing the kinetic changes in the normalized Ca^2+^ transients before and after carbachol, and after carbachol plus atropine. Means ± SEM are represented as the solid horizontal lines. ^*^p < 0.01, *N* = 5 hearts.

To assess the cholinergic regulation of contractility, we examined the properties of Ca^2+^ transients across the ventricular wall in the presence of a cholinergic agonist and antagonist (Table 2). Figure 8 illustrates normalized Ca^2+^ transients recorded simultaneously at both the endocardial (Figure 8A) and epicardial (Figure 8B) layer with carbachol and carbachol and atropine. Perfusion of the hearts with 5 μM carbachol induced a minor but statistically significant decrease in the amplitude of the Ca^2+^ transients by 3.5 ± 0.35% in the endocardium, and 7.5 ± 0.58% in epicardium (Figure 8C). However, this effect was smaller than the increase in the amplitude of Ca^2+^ transients observed when hearts were perfused with isoproterenol. The effects of carbachol on various kinetic parameters of the Ca^2+^ transients are summarized (*N* = 5 hearts) in Figure 8D. Although, carbachol did not significantly change the normalized rise time in endocardium or epicardium, the time to peak of the Ca^2+^ transients were significantly increased in endocardium (5.5 ± 1.17%) but not in epicardium (2.5 ± 1.83%). Carbachol induced a significant increase in the half duration (endocardium 1.9 ± 0.3%; epicardium 3.0 ± 0.57%) and fall time of the Ca^2+^ transients (endocardium 3.3 ± 0.69%; epicardium 4.1 ± 1.07%). Although the changes in the fall times are significantly different, they were smaller than the kinetic effects observed with isoproterenol (Figure 2). Thus, we conclude that perfusion with carbachol did not have a large impact on the amplitude and the kinetics of endocardial and epicardial Ca^2+^ transients, compared to those observed with the perfusion of isoproterenol. When the hearts were perfused with both agonist and antagonist (5 μM carbachol plus 40 μM atropine) it was possible to observe significant values above the control, for rise time (epicardium 5.3 ± 3.14%), time to peak (endocardium 6.7 ± 1.18%; epicardium 6.5 ± 2.01%), half duration (endocardium 9.4 ± 0.34%; epicardium 10.3 ± 0.64%) and fall time (endocardium 4.2 ± 0.74%; epicardium 6.8 ± 1.29%).

**TABLE 2:** Carbachol effects on Ca^2+^ transient kinetics and AP durations.

**Carbachol effects on Ca^2+^ transient kinetics**				
**Condition**	**RT (ms)**	**TP (ms)**	**HD (ms)**	**FT (ms)**
Endo Control	8.32 ± 0.06 (*n* = 676)	12.07 ± 0.08 (*n* = 734)	54.37 ± 0.25 (*n* = 832)	76.45 ± 0.30 (*n* = 764)
Endo CCh	8.06 ± 0.06 (*n* = 706)^*^	12.63 ± 0.08 (*n* = 738)^*^	55.49 ± 0.29 (*n* = 808)^*^	79.34 ± 0.34 (*n* = 806)^*^
Endo CCh + Atropine	8.75 ± 0.05 (*n* = 545)^*^	13.08 ± 0.10 (*n* = 697)^*^	63.27 ± 0.25 (*n* = 586)^*^	82.80 ± 0.36 (*n* = 583)^*^
Epi Control	6.78 ± 0.07 (*n* = 302)	11.70 ± 0.12 (*n* = 367)	53.97 ± 0.25 (*n* = 462)	78.47 ± 0.39 (*n* = 456)
Epi CCh	7.05 ± 0.07 (*n* = 321)^*^	11.83 ± 0.14 (*n* = 341)	55.32 ± 0.19 (*n* = 520)^*^	81.26 ± 0.43 (*n* = 448)^*^
Epi CCh + Atropine	7.78 ± 0.15 (*n* = 93)^*^	12.16 ± 0.17 (*n* = 357)	58.96 ± 0.23 (*n* = 368)^*^	84.90 ± 0.61 (*n* = 347)^*^

**Carbachol effects on AP durations**				

**Condition**	**APD30 (ms)**	**APD half phase 2 (ms)**	**APD90 (ms)**	

Endo Control	7.41 ± 0.14 (*n* = 264)	81.53 ± 0.31 (*n* = 239)	82.61 ± 0.41 (*n* = 269)	
Endo CCh	6.88 ± 0.11 (*n* = 269)^*^	52.65 ± 0.18 (*n* = 211)^*^	65.65 ± 0.20 (*n* = 240)^*^	
Endo CCh + Atropine	8.69 ± 0.12 (*n* = 440)^*^	67.42 ± 0.16 (*n* = 443)^*^	82.47 ± 0.22 (*n* = 443)	
Epi Control	3.24 ± 0.11 (*n* = 425)	68.90 ± 0.15 (*n* = 581)	81.62 ± 0.18 (*n* = 426)	
Epi CCh	3.22 ± 0.12 (*n* = 442)	52.15 ± 0.19 (*n* = 544)^*^	65.89 ± 0.27 (*n* = 405)^*^	
Epi CCh + Atropine	3.08 ± 0.27 (*n* = 612)^*^	66.05 ± 0.17 (*n* = 628)^*^	79.46 ± 0.18 (*n* = 629)^*^	

Endo, endocardium; Epi, epicardium; CCh, carbachol; RT, rise time; TP, time to peak; HD, half duration; FT, fall time. APD, AP durations measured at 30, half phase 2, and 90% repolarization. ^*^Kolmogorov–Smirnov test, *p* < 0.01, *N* = 5.

**FIGURE 9:**
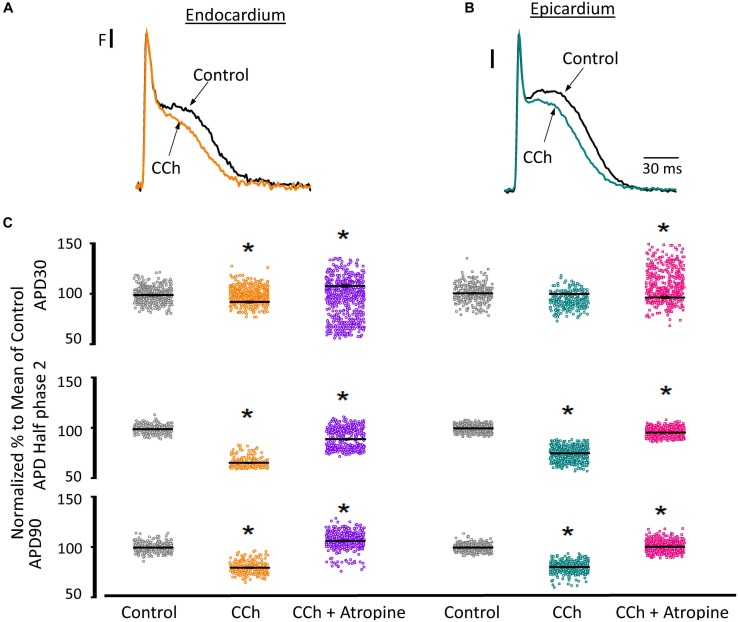
Cholinergic stimulation modifies overall AP morphology in endocardium and epicardium. **(A)** Normalized APs recorded optically with Di-8-ANEPPS from the endocardium and **(B)** epicardium before and after perfusion with 5 μM carbachol (CCh). **(C)** Summary of the normalized APD30, APD half phase 2 and APD90 changes from the endocardium (circles) and epicardium (squares), before and after carbachol, and after carbachol plus atropine. Means ± SEM are represented as the solid horizontal lines. ^*^p < 0.01, *N* = 5 hearts.

**FIGURE 10:**
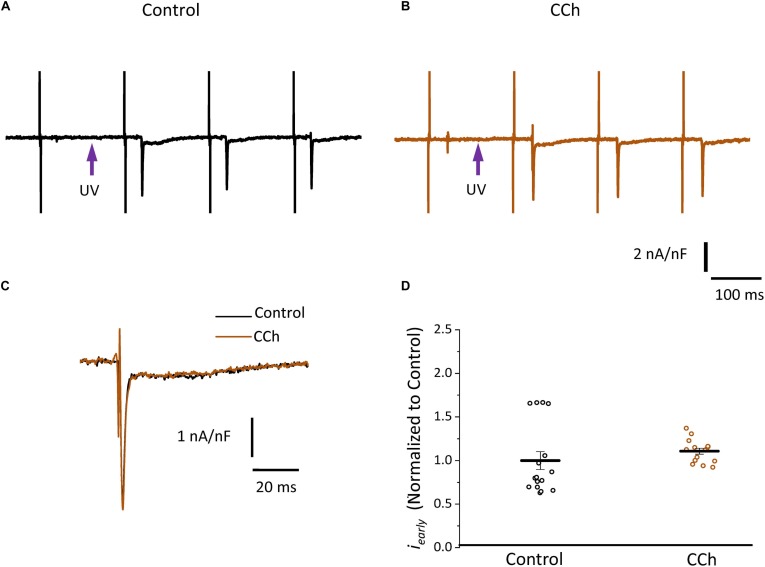
Cholinergic stimulation does not modify L-type Ca^2+^ currents in the absence of a sympathetic drive. **(A)** Representative traces of Ca^2+^-driven currents recorded after the local photolysis of nifedipine using the Loose Patch Photolysis technique showing *i_early_* and *i_late_* components under control conditions and **(B)** in the presence of 5 μM carbachol (CCh). **(C)** Overlapping traces of Ca^2+^-driven currents before (black) and after cholinergic stimulation (brown). **(D)** Normalized data of the peak values of *i_early_*, before and after carbachol.

Since the effects induced by carbachol in the contractility were minor, we decided to explore how the activation of a parasympathetic pathway affects the electrophysiological behavior of the endocardial and the epicardial layers. In Table 2 and Figure 9 we compared ventricular APs optically recorded in the endocardium (Figure 9A) and epicardium (Figure 9B). The perfusion with 5 μM carbachol produced significant changes in the AP morphology. Figure 9C summarizes different endocardial and epicardial AP parameters from five hearts. Carbachol did not alter APD30 in epicardium, but had a very modest effect on the endocardial layer (6.9 ± 1.16%). Interestingly, the APD half phase 2 (endocardium 33.4 ± 0.62%; epicardium 24.9 ± 0.42%) and APD90 (endocardium 20.1 ± 0.68%; epicardium 19.4 ± 0.43%), were significantly reduced by the muscarinic stimulation. An interesting effect by the addition of atropine in the presence of carbachol was the recovery of the tested parameters. The APD half phase 2 in endocardium recovered to 90.0 ± 1.12% and the epicardium to 95.7 ± 0.29%. The APD90 for endocardium and epicardium recovered 106.6 ± 0.67% and 100.8 ± 0.26% respectively, from the control values. This suggests that atropine, aside from blocking carbachol’s action, may also be blocking an intrinsic, existing cholinergic tone in the isolated heart that modulates the time course of the ventricular AP. Moreover, the fact that carbachol clearly modified both APD half phase 2 and APD90 without changing the kinetic properties of Ca^2+^ transients indicates that the Ca^2+^ influx that triggers Ca^2+^ induced Ca^2+^ release may not occur during phase 2. To further explore this possibility, we then evaluated the properties of Ca^2+^ currents driven by its own AP, when the heart was exposed to a parasympathetic drive. Indeed, the decrease of APD half phase 2 with carbachol could be due to a decrease in the amplitude of L-type Ca^2+^ currents. For example, a reduction in the L-type Ca^2+^ current will lead to a decrease in the Ca^2+^ released from the SR. In turn, this will result in less activation of the NCX in its forward mode. To test this scenario, we evaluated the effect of carbachol on the time course of the L-type Ca^2+^ current during an evoked AP. In Figures 10A,B, we recorded the epicardial Ca^2+^-driven currents that were activated by the photolysis of nifedipine before and after carbachol perfusion. Figure 10C shows the superposition of two Ca^2+^-driven current traces before and after the carbachol perfusion. The Ca^2+^-driven currents displayed an *i_early_* and *i_late_* component, but they were not affected by the presence of carbachol. Figure 10D shows that carbachol did not significantly modify the amplitude of *i_early_*.

**FIGURE 11:**
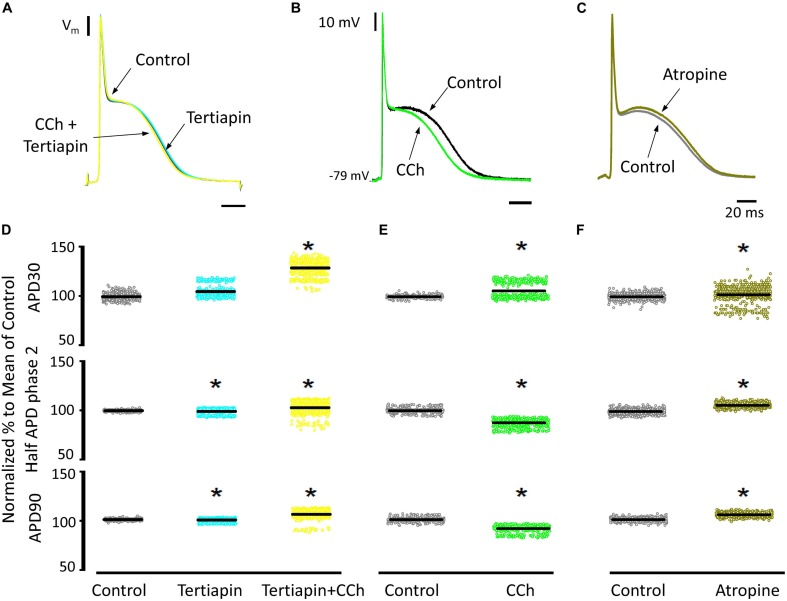
Cholinergic stimulation acts via *I*_KACh_ in ventricle. **(A)** APs recorded from the epicardium using glass microelectrodes before (black), with 100 nM tertiapin (cyan) and after tertiapin with 5 μM carbachol (CCh; yellow). **(B)** Epicardial APs with 5 μM carbachol (green) after 20 min washout from tertiapin + carbachol (black). **(C)** Effect of 40 μM atropine (dark green) on the time course of epicardial APs. **(D)** Data summarizing epicardial APD30, APD half of phase 2, and APD90 of the normalized APs before (gray) and after 100 nM tertiapin followed by the perfusion of 5 μM carbachol plus tertiapin. **(E)** APD30, APD half phase 2, and APD90 after a 20 min washout from tertiapin + carbachol (gray) and then perfusion with 5 μM carbachol (green). **(F)** APD30, APD half phase 2, and APD90 after perfusion of 40 μM atropine. Means ± SEM are represented as the solid horizontal lines; ^*^p < 0.01, *N* = 5 hearts.

Since carbachol did not induce changes in the amplitude of L-type Ca^2+^ currents, we examined if a repolarizing outward current (associated with the cholinergic stimulation) was involved in the shortening of the APD half phase 2. Although it is well established in the atrium that the current of an acetylcholine (ACh) activated potassium channel, *I*_KACh_, can severely affect the AP repolarization, the role of this current in the ventricle is still a matter of debate. The role of the *I*_KACh_ was evaluated by perfusing the hearts with tertiapin, a bee venom that can block with high affinity GIRK1/GIRK2 hetero-tetramers (Hashimoto et al., 2006). GIRK1/GIRK2 hetero-tetramers form the pore of the inward rectifying K^+^ channels responsible for *I*_KACh_. The perfusion of tertiapin prevented the carbachol shortening of the AP in the epicardium Table 3 and Figure 11. Figure 11A illustrates that in the presence of tertiapin, 5 μM carbachol is not able to produce any visible change in the kinetics of the electrically recorded APs. However, in the absence of tertiapin, 5 μM carbachol modified APD half phase 2 and APD90 (Figure 11B). The summarized epicardial AP data (Figure 11D; *N* = 5 hearts) shows tertiapin induced statistically significant differences in all of the AP phases (5.2 ± 0.38% for APD30, 0.8 ± 0.56% for APD half phase 2 and 0.5 ± 0.02% for APD90). Although these differences are statistically significant, the magnitude of the change is so small (see Table 3) that we do not consider that these changes will have any physiological effect. Interestingly, in the presence of tertiapin, carbachol was not able to induce the same shortening effect, as in the absence of tertiapin, on the total AP duration (APD half phase 2 and APD90, 3 ± 0.71% and 5.3 ± 0.19%, respectively). To corroborate that tertiapin was blocking the carbachol effect, we perfused the heart with carbachol after tertiapin was washed out (Figures 11B,E). Carbachol significantly modified APD30 (5.9 ± 0.42%) above control and decreased both APD half phase 2 (12.4 ± 0.22%) and APD90 (9.9 ± 0.19%). These results were consistent with the ones reported in Figure 9C. Overall, these results suggest that the AP shortening produced by a cholinergic stimulation is due to the activation of *I*_KACh_ instead of a Ca^2+^-driven mechanism. Finally, we evaluated if in the perfused heart, there is a tonic release of acetylcholine from the postganglionic parasympathetic terminals located in the ventricular wall. Figure 11C shows that 40 μM atropine prolongs the late repolarization of the epicardial AP. Indeed, although the increase in APD half phase 2 (6.0 ± 0.11%) and APD90 (4.9 ± 0.02%) are not prominent, the increase is statistically significant and reflects a change >5 ms (Table 3 and Figure 11F). This result indicates that it is likely that an intrinsic parasympathetic tone is present in the isolated heart and can slightly decrease the duration of ventricular APs.

**TABLE 3:** Tertiapin, carbachol, and atropine effects on AP durations.

**Condition**	**APD30 (ms)**	**APD half phase 2 (ms)**	**APD90 (ms)**
Epi Control	2.47 ± 0.01 (*n* = 650)	48.63 ± 0.10 (*n* = 650)	60.72 ± 0.08 (*n* = 649)
Epi Tertiapin	2.62 ± 0.01 (*n* = 650)^*^	48.36 ± 0.15 (*n* = 643)^*^	60.46 ± 0.12 (*n* = 650)^*^
Epi Tertiapin + CCh	3.24 ± 0.02 (*n* = 602)^*^	50.21 ± 0.19 (*n* = 642)^*^	64.03 ± 0.15 (*n* = 641)^*^
Epi Control (after Tertiapin + CCh washout)	2.39 ± 0.01 (*n* = 423)	53.89 ± 0.27 (*n* = 582)	67.18 ± 0.28 (*n* = 582)
Epi CCh	2.64 ± 0.02 (*n* = 393)^*^	47.54 ± 0.18 (*n* = 432)^*^	62.08 ± 0.27 (*n* = 431)^*^
Epi Control	2.58 ± 0.01 (*n* = 720)	60.71 ± 0.41 (*n* = 719)	70.19 ± 0.45 (*n* = 720)
Epi Atropine	2.61 ± 0.01 (*n* = 566)^*^	67.33 ± 0.45 (*n* = 566)^*^	75.19 ± 0.58 (*n* = 566)^*^

Epi, epicardium; CCh, carbachol. APD, AP durations measured at 30, half phase 2, and 90% repolarization. ^*^Kolmogorov–Smirnov test, *p* < 0.01, *N* = 5.

## Discussion

Although, there is a substantial body of experimental information on sympathetic and parasympathetic regulation of the electrical and mechanical properties of the heart, there is no conclusive evidence of how autonomic driven changes affects the relationship between contractility and excitability in the mouse heart. Neither is it known how sympathetic and parasympathetic nervous system regulate those properties across the ventricular wall. In this study, we investigated how either β-adrenergic or muscarinic cholinergic agonists regulate the time course of ventricular APs and how these electrical changes correlate with the left ventricular pressure, Ca^2+^ transients, and Ca^2+^-driven currents at the whole-heart level. Our results indicate that in the mouse heart the influx of Ca^2+^ that triggers the ECC happens during AP phase 1 and not phase 2, contrary to what occurs in larger mammals (Boyett, 1986; Antzelevitch et al., 1991; Qu et al., 2013). Indeed, in isolated mice hearts, sympathetic stimulation reduced the rate of repolarization and increased the amplitude of epicardial Ca^2+^ currents. The increase in Ca^2+^ influx drives an increase in the amplitude of Ca^2+^ transients both in the endocardium and the epicardium that finally produces an increase in the developed pressure. In contrast, parasympathetic stimulation dramatically reduced the duration of phase 2 without inducing major changes in Ca^2+^ currents, Ca^2+^ transients and developed pressure indicating that the Ca^2+^ signaling involved in controlling the ECC does not occur during the AP phase 2.

### β-Adrenergic Stimulation Across the Ventricular Wall Regulate Ca^2+^-Driven Mechanisms

It is well known that β-adrenergic stimulation will accelerate the heart rate (chronotropism), and the higher systemic demands will be met by increasing the systolic pressure (inotropism) as well as the rate of relaxation (lusitropism) of the developed pressure. In our mouse model, exposure to isoproterenol generated an increase in the left ventricular developed pressure (Figure 1). This increase in inotropism resulted because of an increase in the amplitude of cytosolic Ca^2+^ transients in both endocardial and epicardial layers (Figure 2). Although we found no significant changes in the expression of β-adrenergic and muscarinic receptors between the endocardial and the epicardial layer, there were differences in the expression of downstream targets (Figure 3). For example, there was a higher expression of SERCA in the epicardial layer. This molecular difference correlates well with the recorded shorter half durations and fall times of Ca^2+^ transients with isoproterenol, in this layer (Figure 2 and Table 1). In addition, the higher expression of RyR2 in the epicardium can explain the faster activation of Ca^2+^ transients and the larger β-adrenergic modulation of the rise time in this layer (Mattiazzi et al., 2015).

There are intrinsic functional differences in the sympathetic regulation of the endocardial and epicardial APs. The differences are imposed by the differential expression of ionic channels in endocardium versus epicardium as reflected by their respective AP morphologies (Antzelevitch et al., 1991; Antzelevitch and Fish, 2001; Abd Allah et al., 2012). Indeed, in Figure 3 we showed a higher expression of Kv4.3 in the epicardial layer. The higher expression of this K^+^ channel explains the faster rate of repolarization of AP phase 1 in epicardium. In addition, β-adrenergic stimulation affected the AP’s morphology by increasing the duration of the AP phase 1 and the amplitude of phase 2 across the ventricular wall (Figure 4). One possibility that may explain the increase in the AP phase 1 duration is a reduction in the amplitude of a repolarizing current, such as *I*_to_. There are few reports of β-adrenergic regulation of *I*_to_. Some experimental evidence (Nakayama et al., 1989; Setién et al., 2013) showed that the catecholamine-induced phosphorylation of the transient outward current (*I*_to_) components increased the amplitude of this fast repolarizing current. This possibility is unlikely to occur in our experimental conditions. Increase of *I*_to_, would accelerate instead of slowing the repolarization rate of phase 1. In contrast, a reduction of the amplitude of *I*_to_ by norepinephrine and alpha adrenoceptor stimulation has been reported in healthy rat ventricular myocytes (Ravens et al., 1989; Gallego and Casis, 2001) which may explain our results. However, this is very unlikely in our experiments because isoproterenol is a β-adrenergic and not an α-adrenergic agonist at the concentrations used here. In either scenario, the increase in the duration of phase 1 (APD30) is most likely a consequence of the competition between *I*_to_ and the inward L-type Ca^2+^ current (López Alarcón et al., 2019). Indeed, a β-adrenergic increase of the L-type Ca^2+^ current (i.e., by PKA phosphorylation) during phase 1 will maintain a depolarization of this phase for a longer time, increasing APD30. Additionally, in the presence of nifedipine, the effect of isoproterenol on APD30 was smaller (Figure 5A). Altogether, these results support the idea that the increase in duration of AP phase 1 correlates well with larger L-type Ca^2+^ currents under isoproterenol stimulation. These findings agree with previous experiments from our group in which we showed that the transmembrane Ca^2+^ influx that triggers Ca^2+^-induced Ca^2+^-released (CICR) enters during phase 1 (Ramos-Franco et al., 2016).

A final proof of concept was to demonstrate that isoproterenol mediates an increase of the amplitude of the L-type Ca^2+^ current during phase 1. Indeed, we corroborated that under β-adrenergic stimulation there is an increase in the amplitude of the L-type Ca^2+^ current during phase 1 (Figure 5). This could result in (1) the decrease of the rate of AP repolarization during phase 1, and (2) the depolarization induced by the increase in the L-type Ca^2+^ current will further activate L-type Ca^2+^ channels with the enlargement of this Ca^2+^ current. Interestingly, in the presence of a submaximal concentration of nifedipine, isoproterenol increased the duration of phase 2. This effect can be attributed to an increase in the amplitude of the Ca^2+^ transients as shown in Figure 2. The most likely possibility is that an increase in the amplitude of the Ca^2+^ transient will induce a larger activation of the NCX in the forward mode as previously suggested (Ferreiro et al., 2012). The validity of this hypothesis was assessed in Figure 5B where we found an increase in the fast and early inward current (*i_early_*) through the L-type Ca^2+^ channels and a significant increase in the amplitude of the late NCX current as reflected by *i_late_*. These results suggest that changes in the electrical excitability induced by the β-adrenergic stimulation across the ventricular wall are highly mediated through a Ca^2+^-driven mechanism.

As we showed, the sympathetic branch of the ANS can induce mechanical changes in the intact heart by altering the underlying plasma membrane Ca^2+^ currents and consequently, Ca^2+^ release from the SR. However, PKA activation can affect other targets. For example, PLN (Toyofuku et al., 1993; Frank and Kranias, 2000), a protein that regulates the activity of SERCA can be phosphorylated on residue S16 (Chu et al., 2000; Valverde et al., 2006). This phosphorylation will relieve the inhibitory effect PLN has on SERCA, that will result in increased contractility (Luo et al., 1994). On the other hand, RyR2 can be phosphorylated by PKA in the residues S2030 (Huke and Bers, 2008) and S2808 (Obayashi et al., 2006; Chen-Izu et al., 2007). This phosphorylation can increase the RyR2 open probability. However, it is not fully established that the RyR2 phosphorylation by itself will increase contractility mostly because of the changes produced in the intra-SR Ca^2+^ content as a consequence of the interplay between release and recapture (Eisner et al., 2004). Although it is accepted that the phosphorylation of these targets will modify Ca^2+^ release from the SR, the timing of the PKA induced phosphorylation is unknown. Specifically, it is unclear which phosphorylated target will have a faster effect on the ECC process. Figure 6 clearly showed that under a global increase in cAMP concentration the L-type Ca^2+^ current is the first transport mechanism that is activated by PKA. However, this may not be the case *in vivo*, due to the documented compartmentation of cAMP resulting from localized cyclic nucleotide phosphodiesterase (PDE) activity (Nikolaev et al., 2006; Leroy et al., 2008). In our experimental conditions (cAMP cell loading), we assumed a homogeneous cellular distribution of cAMP. Based on this, we propose that PKA phosphorylation of the L-type Ca^2+^ channels has a dual effect. Immediately after phosphorylation, the increase in L-type Ca^2+^ current will increase Ca^2+^ release from the SR. Consequently, the increase in the intracellular Ca^2+^ will promote a larger uptake of Ca^2+^ into the SR mediated by SERCA. Finally, the phosphorylation of PLN will induce a later increase in the intra-SR Ca^2+^ content that will further increase SR Ca^2+^ release.

### Cholinergic Stimulation Across the Ventricular Wall Did Not Alter Ca^2+^ Dynamics

Although the sympathetic branch of the ANS produced mechanical changes in the intact heart by altering the underlying Ca^2+^ currents, the cholinergic stimulation did not. Interestingly, the left ventricular pressure showed no changes when exposed to carbachol (Figure 7). However, there were minor changes in the amplitude of the Ca^2+^ transients in both endocardium and epicardium (Figure 8). Carbachol induced some statistically significant changes in most of the kinetic parameters of Ca^2+^ transients, but these changes were never larger than 4% (Figure 8D). This suggest that although parasympathetic regulation has a negative inotropic effect on the cardiac function at the whole animal level, in the Langendorff perfused heart, we were unable to observe any substantial effect in contractility. One difference between the intact animal and the isolated perfused heart relies on the fact that, in the perfused heart, we do not have an intrinsic systemic sympathetic drive. Indeed, in the intact animal, the binding of acetylcholine to muscarinic receptors will activate a *G*_i_ protein that will exert an inhibitory effect on AC, antagonizing the stimulatory effect of the β-AR coupled to a *G*_s_ protein. However, in the isolated heart, if *G*_s_ is not activating AC, the activation of a *G*_i_ will not produce changes in the intracellular cAMP levels. Finally, this would explain why we did not observe any major negative inotropic action of carbachol in the perfused heart in the absence of a sympathetic drive.

### Cholinergic Stimulation Modifies Endocardial and Epicardial Cardiac Excitability

Recently, it was shown that vagal innervation exists in the ventricle, but it is 80% less innervated than atria (Coote, 2013). Parasympathetic action on the AP’s waveform has been mostly evaluated in atrial and nodal myocytes (sinus and atrio-ventricular). In both cell types, acetylcholine induces an increase in the AP repolarization rate mediated by a K^+^ current activated by acetylcholine (*I*_KACh_; Engelstein et al., 1994; Corey et al., 1998; Wickman et al., 1999; Choisy et al., 2012). There was a general belief that this mechanism is absent in the ventricle (Coote, 2013). Recently, immunofluorescence studies have shown the presence of G-protein-coupled inward rectifier K^+^ proteins in mouse, rat, and human ventricle (Liang et al., 2014). Here, we found that, in a mouse ventricle, carbachol had a significant effect on AP repolarization rate (Figure 9). The APD half phase 2 and the APD90 were reduced in both the endocardial and the epicardial layers, with a modest effect on APD30. Furthermore, atropine, an antagonist of M2 muscarinic receptors, was able to prevent the effect of carbachol indicating that this effect was mediated by a muscarinic receptor.

Interestingly, Litovsky and Antzelevitch (1990) reported that acetylcholine had little if any effect in canine ventricular endocardium but a concentration-dependent biphasic effect in epicardium. Specifically, acetylcholine induced an APD50 prolongation at 0.1 and 1 μM and a reduction at 10 μM. The discrepancy with our findings could be explained by the difference in species (canine vs. murine) and tissue preparation (ventricular wedges vs. whole heart). Another difference is that these authors found no significant effects on APD30 in either layer (Litovsky and Antzelevitch, 1990). Even though our APD30 results showed a significant difference only for the endocardium, the change induced by carbachol was of 530 ± 250 μs (Table 2). This difference is 4 times smaller than the increase that we observed in APD30 when the heart was perfused with isoproterenol (Table 1). The fact that carbachol had a profound effect on the AP morphology but minor effects on contractility and Ca^2+^ currents, supports the idea that Ca^2+^influx, that determine the CICR, does not occur during AP phase 2.

### Cholinergic Stimulation Across the Ventricular Wall Was Mediated via *I*_KACh_

In general, there are two main hypotheses to explain the muscarinic actions on AP repolarization rate: (1) inhibitory effect on the Ca^2+^ influx through L-type Ca^2+^ channels or (2) an agonistic effect on a cationic outward current. In principle, the muscarinic effect on the L-type Ca^2+^ is very unlikely because, as we showed, there were no effects on the developed pressure and very minor effects (3.5 ± 0.35% in the endocardium, and 7.5 ± 0.58% in epicardium) on the amplitude of the Ca^2+^ transients in the presence of carbachol. Even more, this first hypothesis was discarded when epicardial Ca^2+^ currents were not affected by carbachol (Figure 10). The second scenario where the increase in the rate of AP repolarization is mediated by the activation of an outward K^+^ current was tested in Figure 11. When the hearts were perfused simultaneously with carbachol and the *I*_KACh_ blocker (tertiapin) a small shortening of the total duration of the AP was observed. Moreover, this apparent blocking effect was corroborated when the same hearts were perfused with carbachol after tertiapin was washed out (Figure 11). Together these results suggest that, in the mouse ventricle, cholinergic-mediated changes in the AP morphology are driven by *I*_KACh_.

## Conclusion

In conclusion, our results indicate that: (1) in both endocardium and epicardium, the increase in contractility by isoproterenol was driven by an increase in the amplitude of intracellular Ca^2+^ currents triggered during the AP phase 1; (2) PKA phosphorylates L-type Ca^2+^ channels before the SR Ca^2+^ release activates NCX current; (3) cholinergic stimulation by carbachol decreased the duration of the late AP repolarization. However, this cholinergic stimulation did not substantially modify *per se* the intracellular Ca^2+^ signals when compared with β-adrenergic stimulation; (4) cholinergic stimulation decreased the total duration of the ventricular AP through activation of *I*_KACh_.

In summary, the results presented here demonstrate that, in a mouse heart, β-adrenergic input acts across the ventricular wall by modulating the L-type Ca^2+^ currents that occur in phase 1 but not phase 2 of the APs. In contrast, a cholinergic input does not directly modulate Ca^2+^ dynamics, but rather alters inward rectifier potassium channels in the isolated heart.

## Ethics Statement

Mice were maintained in accordance to the National Institutes of Health Guide for the Care and Use of Laboratory Animals (NIH Publication No. 85–23, Revised 1996) and the Institutional Animal Care and Use Committee guidelines of the University of California Merced (Protocol # 2008–201).

## Author Contributions

AE and JR-F designed the research. YA-S, AR, MA, AE, and JR-F performed the research. YA-S, AR, AE, and JR-F analyzed the data. YA-S, AE, and JR-F wrote the manuscript.

## Conflict of Interest Statement

The authors declare that the research was conducted in the absence of any commercial or financial relationships that could be construed as a potential conflict of interest.

## References

[B1] Abd Allah E. S. H. Aslanidi O. V. Tellez J. O. Yanni J. Billeter R. Zhang H. (2012). Postnatal development of transmural gradients in expression of ion channels and Ca2+-handling proteins in the ventricle. *J. Mol. Cell. Cardiol.* 53 145–155. 10.1016/j.yjmcc.2012.04.004 22537893

[B2] Aguilar-Sanchez Y. Fainstein D. Mejia-Alvarez R. Escobar A. L. (2017). Local field fluorescence microscopy: imaging cellular signals in intact hearts. *J. Vis. Exp* 121:e55202. 10.3791/55202 28362405 PMC5408857

[B3] Antzelevitch C. Fish J. (2001). Electrical heterogeneity within the ventricular wall. *Basic Res. Cardiol.* 96 517–527. 11770069 10.1007/s003950170002

[B4] Antzelevitch C. Sicouri S. Litovsky S. H. Lukas A. Krishnan S. C. Di Diego J. M. (1991). Heterogeneity within the ventricular wall. *Electrophysiology and pharmacology of epicardial, endocardial, and M cells*. *Circ. Res.* 69 1427–1449. 1659499 10.1161/01.res.69.6.1427

[B5] Boyett M. R. (1986). Current concepts on the electrophysiology of the myocardium. *J. Perinat. Med.* 14 349–354. 2434639 10.1515/jpme.1986.14.6.349

[B6] Breitwieser G. E. Szabo G. (1988). Mechanism of muscarinic receptor-induced K+ channel activation as revealed by hydrolysis-resistant GTP analogues. *J. Gen. Physiol.* 91 469–493. 2455765 10.1085/jgp.91.4.469 PMC2216147

[B7] Brum G. Osterrieder W. Trautwein W. (1984). Beta-adrenergic increase in the calcium conductance of cardiac myocytes studied with the patch clamp. *Pflugers Arch.* 401 111–118. 6089094 10.1007/BF00583870

[B8] Chen-Izu Y. Ward C. W. Stark W. Banyasz T. Sumandea M. P. Balke C. W. (2007). Phosphorylation of RyR2 and shortening of RyR2 cluster spacing in spontaneously hypertensive rat with heart failure. *Am. J. Physiol. Heart Circ. Physiol.* 293 H2409–H2417. 10.1152/ajpheart.00562.2007 17630346

[B9] Choisy S. C. M. James A. F. Hancox J. C. (2012). Acute desensitization of acetylcholine and endothelin-1 activated inward rectifier K+ current in myocytes from the cardiac atrioventricular node. *Biochem. Biophys. Res. Commun.* 423 496–502. 10.1016/j.bbrc.2012.05.148 22683635 PMC3400056

[B10] Chu G. Lester J. W. Young K. B. Luo W. Zhai J. Kranias E. G. (2000). A single site (Ser16) phosphorylation in phospholamban is sufficient in mediating its maximal cardiac responses to beta -agonists. *J. Biol. Chem.* 275 38938–38943. 10.1074/jbc.M004079200 10988285

[B11] Clapham D. E. Kim D. (1989). G protein activation mechanisms of the cardiac K+ channel, iK.A*Ch*. *Soc. Gen. Physiol. Ser.* 44 55–68. 2675324

[B12] Cohn J. N. (1989). Sympathetic nervous system activity and the heart. *Am. J. Hypertens* 2 353S–356S. 2532019

[B13] Collins J. H. Kranias E. G. Reeves A. S. Bilezikjian L. M. Schwartz A. (1981). Isolation of phospholamban and a second proteolipid component from canine cardiac sarcoplasmic reticulum. *Biochem. Biophys. Res. Commun.* 99 796–803. 6454414 10.1016/0006-291x(81)91235-3

[B14] Coote J. H. (2013). Myths and realities of the cardiac vagus. *J. Physiol.* 591 4073–4085. 10.1113/jphysiol.2013.257758 23878363 PMC3779103

[B15] Corey S. Krapivinsky G. Krapivinsky L. Clapham D. E. (1998). Number and stoichiometry of subunits in the native atrial G-protein-gated K+ channel, IKACh. *J. Biol. Chem.* 273 5271–5278. 9478984 10.1074/jbc.273.9.5271

[B16] Dilly K. W. Rossow C. F. Votaw V. S. Meabon J. S. Cabarrus J. L. Santana L. F. (2006). Mechanisms underlying variations in excitation-contraction coupling across the mouse left ventricular free wall: heterogeneous EC coupling in heart. *J. Physiol.* 572 227–241. 10.1113/jphysiol.2005.102020 16423856 PMC1779645

[B17] Eisner D. A. Díaz M. E. O’Neill S. C. Trafford A. W. (2004). Physiological and pathological modulation of ryanodine receptor function in cardiac muscle. *Cell Calcium* 35 583–589. 10.1016/j.ceca.2004.01.012 15110148

[B18] Engelstein E. D. Lippman N. Stein K. M. Lerman B. B. (1994). Mechanism-specific effects of adenosine on atrial tachycardia. *Circulation* 89 2645–2654. 8205677 10.1161/01.cir.89.6.2645

[B19] Evans D. B. (1986). Modulation of cAMP: mechanism for positive inotropic action. *J. Cardiovasc. Pharmacol.* 8(Suppl. 9), S22–S29. 2433539

[B20] Ferreiro M. Petrosky A. D. Escobar A. L. (2012). Intracellular Ca^2+^ release underlies the development of phase 2 in mouse ventricular action potentials. *Am. J. Physiol. Heart Circ. Physiol.* 302 H1160–H1172. 10.1152/ajpheart.00524.2011 22198177 PMC3311451

[B21] Frank K. Kranias E. G. (2000). Phospholamban and cardiac contractility. *Ann. Med.* 32 572–578. 10.3109/07853890008998837 11127935

[B22] Gallego M. Casis O. (2001). Regulation of cardiac transient outward potassium current by norepinephrine in normal and diabetic rats. *Diabetes Metab. Res. Rev.* 17 304–309. 11544615 10.1002/dmrr.212

[B23] Hashimoto N. Yamashita T. Tsuruzoe N. (2006). Tertiapin, a selective IKACh blocker, terminates atrial fibrillation with selective atrial effective refractory period prolongation. *Pharmacol. Res.* 54 136–141. 10.1016/j.phrs.2006.03.021 16725344

[B24] Hayes J. S. Mayer S. E. (1981). Regulation of guinea pig heart phosphorylase kinase by cAMP, protein kinase, and calcium. *Am. J. Physiol.* 240 E340–E349. 10.1152/ajpendo.1981.240.3.E340 6259950

[B25] Henning R. J. (1992). Vagal stimulation during muscarinic and beta-adrenergic blockade increases atrial contractility and heart rate. *J. Auton. Nerv. Syst.* 40 121–129. 1464693 10.1016/0165-1838(92)90023-a

[B26] Hildebrandt J. D. Sekura R. D. Codina J. Iyengar R. Manclark C. R. Birnbaumer L. (1983). Stimulation and inhibition of adenylyl cyclases mediated by distinct regulatory proteins. *Nature* 302 706–709. 6300694 10.1038/302706a0

[B27] Hiltunen J. O. Laurikainen A. Airaksinen M. S. Saarma M. (2000). GDNF family receptors in the embryonic and postnatal rat heart and reduced cholinergic innervation in mice hearts lacking ret or GFRalpha2. *Dev. Dyn. Off. Publ. Am. Assoc. Anat* 219 28–39. 10.1002/1097-0177(2000)9999:9999<::AID-DVDY1031>3.0.CO;2-P 10974669

[B28] Huke S. Bers D. M. (2008). Ryanodine receptor phosphorylation at Serine 2030, 2808 and 2814 in rat cardiomyocytes. *Biochem. Biophys. Res. Commun.* 376 80–85. 10.1016/j.bbrc.2008.08.084 18755143 PMC2581610

[B29] Katz A. M. (1967). Regulation of cardiac muscle contractility. *J. Gen. Physiol.* 50(Suppl.), 185–196. 10.1085/jgp.50.6.185 PMC2225748 4227923

[B30] Kornyeyev D. Petrosky A. D. Zepeda B. Ferreiro M. Knollmann B. Escobar A. L. (2012). Calsequestrin 2 deletion shortens the refractoriness of Ca2+ release and reduces rate-dependent Ca2+-alternans in intact mouse hearts. *J. Mol. Cell. Cardiol.* 52 21–31. 10.1016/j.yjmcc.2011.09.020 21983287 PMC3687039

[B31] Kornyeyev D. Reyes M. Escobar A. L. (2010). Luminal Ca(2+) content regulates intracellular Ca(2+) release in subepicardial myocytes of intact beating mouse hearts: effect of exogenous buffers. *Am. J. Physiol. Heart Circ. Physiol.* 298 H2138–H2153. 10.1152/ajpheart.00885.2009 20382849 PMC2886618

[B32] Krebs E. G. (1972). Protein kinases. *Curr. Top. Cell. Regul.* 5 99–133. 4358204

[B33] Kurachi Y. Ito H. Sugimoto T. Katada T. Ui M. (1989). Activation of atrial muscarinic K+ channels by low concentrations of beta gamma subunits of rat brain G protein. *Pflugers Arch.* 413 325–327. 2497438 10.1007/BF00583550

[B34] Lee W. C. Shideman F. E. (1959). Role of myocardial catecholamines in cardiac contractility. *Science* 129 967–968. 13646629 10.1126/science.129.3354.967

[B35] Leroy J. Abi-Gerges A. Nikolaev V. O. Richter W. Lechêne P. Mazet J.-L. (2008). Spatiotemporal dynamics of beta-adrenergic cAMP signals and L-type Ca2+ channel regulation in adult rat ventricular myocytes: role of phosphodiesterases. *Circ. Res.* 102 1091–1100. 10.1161/CIRCRESAHA.107.167817 18369156

[B36] Liang B. Nissen J. D. Laursen M. Wang X. Skibsbye L. Hearing M. C. (2014). G-protein-coupled inward rectifier potassium current contributes to ventricular repolarization. *Cardiovasc. Res.* 101 175–184. 10.1093/cvr/cvt240 24148898 PMC3868351

[B37] Lindemann J. P. Watanabe A. M. (1985). Muscarinic cholinergic inhibition of beta-adrenergic stimulation of phospholamban phosphorylation and Ca2+ transport in guinea pig ventricles. *J. Biol. Chem.* 260 13122–13129. 2414274

[B38] Litovsky S. H. Antzelevitch C. (1990). Differences in the electrophysiological response of canine ventricular subendocardium and subepicardium to acetylcholine and isoproterenol. *A direct effect of acetylcholine in ventricular myocardium*. *Circ. Res.* 67 615–627. 2397572 10.1161/01.res.67.3.615

[B39] López Alarcón M. M. Rodríguez de Yurre A. Felice J. I. Medei E. Escobar A. L. (2019). Phase 1 repolarization rate defines Ca2+ dynamics and contractility on intact mouse hearts. *J. Gen. Physiol.* 151 771–785. 10.1085/jgp.201812269 31000581 PMC6571993

[B40] Luo W. Grupp I. L. Harrer J. Ponniah S. Grupp G. Duffy J. J. (1994). Targeted ablation of the phospholamban gene is associated with markedly enhanced myocardial contractility and loss of beta-agonist stimulation. *Circ. Res.* 75 401–409. 8062415 10.1161/01.res.75.3.401

[B41] Marks A. R. (2013). Calcium cycling proteins and heart failure: mechanisms and therapeutics. *J. Clin. Invest.* 123 46–52. 10.1172/JCI62834 23281409 PMC3533269

[B42] Mattiazzi A. Argenziano M. Aguilar-Sanchez Y. Mazzocchi G. Escobar A. L. (2015). Ca2+ Sparks and Ca2+ waves are the subcellular events underlying Ca2+ overload during ischemia and reperfusion in perfused intact hearts. *J. Mol. Cell. Cardiol.* 79 69–78. 10.1016/j.yjmcc.2014.10.011 25451173 PMC4302011

[B43] Mejía-Alvarez R. Manno C. Villalba-Galea C. A. del Valle Fernández L. Costa R. R. Fill M. (2003). Pulsed local-field fluorescence microscopy: a new approach for measuring cellular signals in the beating heart. *Pflugers Arch* 445 747–758. 10.1007/s00424-002-0963-961 12632197

[B44] Mundiña de Weilenmann C. Vittone L. de Cingolani G. Mattiazzi A. (1987). Dissociation between contraction and relaxation: the possible role of phospholamban phosphorylation. *Basic Res. Cardiol.* 82 507–516. 2963614 10.1007/BF01907220

[B45] Nakayama T. Palfrey C. Fozzard H. A. (1989). Modulation of the cardiac transient outward current by catecholamines. *J. Mol. Cell. Cardiol.* 21(Suppl. 1), 109–118. 2471837 10.1016/0022-2828(89)90845-6

[B46] Nerbonne J. M. Kass R. S. (2005). Molecular Physiology of Cardiac Repolarization. *Physiol. Rev.* 85 1205–1253. 10.1152/physrev.00002.2005 16183911

[B47] Nikolaev V. O. Bünemann M. Schmitteckert E. Lohse M. J. Engelhardt S. (2006). Cyclic AMP imaging in adult cardiac myocytes reveals far-reaching beta1-adrenergic but locally confined beta2-adrenergic receptor-mediated signaling. *Circ. Res.* 99 1084–1091. 10.1161/01.RES.0000250046.69918.d5 17038640

[B48] Obayashi M. Xiao B. Stuyvers B. D. Davidoff A. W. Mei J. Chen S. R. W. (2006). Spontaneous diastolic contractions and phosphorylation of the cardiac ryanodine receptor at serine-2808 in congestive heart failure in rat. *Cardiovasc. Res.* 69 140–151. 10.1016/j.cardiores.2005.07.010 16112660

[B49] Osterrieder W. Brum G. Hescheler J. Trautwein W. Flockerzi V. Hofmann F. (1982). Injection of subunits of cyclic AMP-dependent protein kinase into cardiac myocytes modulates Ca2+ current. *Nature* 298 576–578. 6285199 10.1038/298576a0

[B50] Qu Z. Xie L.-H. Olcese R. Karagueuzian H. S. Chen P.-S. Garfinkel A. (2013). Early afterdepolarizations in cardiac myocytes: beyond reduced repolarization reserve. *Cardiovasc. Res.* 99 6–15. 10.1093/cvr/cvt104 23619423 PMC3687754

[B51] Ramos-Franco J. Aguilar-Sanchez Y. Escobar A. L. (2016). Intact heart loose patch photolysis reveals ionic current kinetics during ventricular action potentials. *Circ. Res.* 118 203–215. 10.1161/CIRCRESAHA.115.307399 26565013 PMC4851170

[B52] Ravens U. Wang X. L. Wettwer E. (1989). Alpha adrenoceptor stimulation reduces outward currents in rat ventricular myocytes. *J. Pharmacol. Exp. Ther.* 250 364–370. 2545864

[B53] Rysevaite K. Saburkina I. Pauziene N. Vaitkevicius R. Noujaim S. F. Jalife J. (2011). Immunohistochemical characterization of the intrinsic cardiac neural plexus in whole-mount mouse heart preparations. *Heart Rhythm* 8 731–738. 10.1016/j.hrthm.2011.01.013 21232628 PMC3081960

[B54] Setién R. Alday A. Diaz-Asensio C. Urrutia J. Gallego M. Casis O. (2013). Mechanisms responsible for the trophic effect of beta-adrenoceptors on the I(to) current density in type 1 diabetic rat cardiomyocytes. *Cell. Physiol. Biochem.* 31 25–36. 10.1159/000343346 23343624

[B55] Suko J. Maurer-Fogy I. Plank B. Bertel O. Wyskovsky W. Hohenegger M. (1993). Phosphorylation of serine 2843 in ryanodine receptor-calcium release channel of skeletal muscle by cAMP-, cGMP- and CaM-dependent protein kinase. *Biochim. Biophys. Acta* 1175 193–206. 8380342 10.1016/0167-4889(93)90023-i

[B56] Toyofuku T. Kurzydlowski K. Tada M. MacLennan D. H. (1993). Identification of regions in the Ca(2+)-ATPase of sarcoplasmic reticulum that affect functional association with phospholamban. *J. Biol. Chem.* 268 2809–2815. 8428955

[B57] Valdivia H. H. Kaplan J. H. Ellis-Davies G. C. Lederer W. J. (1995). Rapid adaptation of cardiac ryanodine receptors: modulation by Mg2+ and phosphorylation. *Science* 267 1997–2000. 7701323 10.1126/science.7701323 PMC4242209

[B58] Valverde C. A. Kornyeyev D. Ferreiro M. Petrosky A. D. Mattiazzi A. Escobar A. L. (2010). Transient Ca2+ depletion of the sarcoplasmic reticulum at the onset of reperfusion. *Cardiovasc. Res.* 85 671–680. 10.1093/cvr/cvp371 19920131 PMC2819836

[B59] Valverde C. A. Mundiña-Weilenmann C. Reyes M. Kranias E. G. Escobar A. L. Mattiazzi A. (2006). Phospholamban phosphorylation sites enhance the recovery of intracellular Ca2+ after perfusion arrest in isolated, perfused mouse heart. *Cardiovasc. Res.* 70 335–345. 10.1016/j.cardiores.2006.01.018 16516179

[B60] Wickman K. Krapivinsky G. Corey S. Kennedy M. Nemec J. Medina I. (1999). Structure, G protein activation, and functional relevance of the cardiac G protein-gated K+ channel, IKACh. *Ann. N. Y. Acad. Sci.* 868 386–398. 10414308 10.1111/j.1749-6632.1999.tb11300.x

