# Supplementary material for: Transmural Autonomic Regulation of Cardiac Contractility at the Intact Heart Level
Source: Front Physiol. 2019 Jul 3;10:773. doi: 10.3389/fphys.2019.00773 (PMC6616252; doi:10.3389/fphys.2019.00773)
Supplement: Supplementary file 1 [file Table_1.docx]

***Supplementary Material***

**Supplementary Table 1**: qPCR primers utilized in this study.

| **Gene** | **Sequence (5’🡪3’)** |
| --- | --- |
| CHRM2 | *Forward*: GGCCCCAGCCATTCTCTTCT |
|  | *Reverse*: TGGTTGGCCACTGGTTCCTT |
| GAPDH | *Forward*: TGCATCCTGCACCACCAACT |
|  | *Reverse*: CTTGGCAGCACCAGTGGATG |
| ADRB1 | *Forward*: GACTTCCGCAAGGCTTTCCA |
|  | *Reverse*: GTCCAGGCTCGAATCGCTGT |
| ADRB2 | *Forward*: TTCTACGTGCCCCTGGTGGT |
|  | *Reverse*: GCAGCCAGCAGAGGGTGAAT |
| CaV1.2 | *Forward*: GGAGAGTCCAGCGAGAAACTCAA |
|  | *Reverse*: CGGCGTTCTCCATCTCCTCTATT |
| NCX1 | *Forward*: GGCTGGGCCTGCTTCATTGT |
|  | *Reverse*: TTTCTGGCCTCCGCCGATAC |
| CASQ2 | *Forward*: GACCGAGTGGTCAGCCTTTC |
|  | *Reverse*: ACAGGTTCGTGGTAATAGAGACA |
| PKAR1a | *Forward*: GAGCCGGGGGATGAGTTCTT |
|  | *Reverse*: GCCCGAGGACGATTCATCAG |
| RyR2 | *Forward*: CCCTTGGGATGCATGAGACA |
|  | *Reverse*: AGCTGCGGCCACGTCTAAAG |
| SERCA2 | *Forward*: CCCGCTGTTTTGCTCGAGTT |
|  | *Reverse*: GTAGATGGCTCGCCCCTCCT |
| PLN | *Forward*: TGACGATCACCGAAGCCAAG |
|  | *Reverse*: CGTGCTTGCTGAGGCATTTC |
| KV4.3 | *Forward*: ACCTGCTGCTCCCGTCGTAG |
|  | *Reverse*: GGCCGTGGTAATCTGGGATG |
